# COVID-19: Auswirkungen auf das zentrale und periphere Nervensystem

**DOI:** 10.1007/s00292-021-00924-x

**Published:** 2021-03-01

**Authors:** N. Ritschel, H. Radbruch, C. Herden, N. Schneider, C. Dittmayer, J. Franz, C. Thomas, G. Silva Boos, A. Pagenstecher, W. Schulz-Schaeffer, C. Stadelmann, M. Glatzel, F. L. Heppner, J. Weis, K. Sohrabi, A. Schänzer, A. Németh, T. Acker, DGNN-Taskforce „CNS-COVID19“, H. Radbruch, C. Herden, A. Pagenstecher, W. Schulz-Schaeffer, C. Stadelmann, T. Acker, H. Radbruch, M. Glatzel, F. L. Heppner, J. Weis, T. Acker, „DEFEAT PANDEMIcs – Neuropathologische Referenzdiagnostik bei COVID-19“

**Affiliations:** 1 Institut für Neuropathologie, Justus-Liebig-Universität Gießen, Arndtstraße 16, 35392 Gießen, Deutschland; 2 Institut für Neuropathologie, Charité – Universitätsmedizin Berlin, Berlin, Deutschland; 3 Institut für Veterinär-Pathologie, Justus-Liebig-Universität Gießen, Gießen, Deutschland; 4 Institut für Medizinische Informatik, Justus-Liebig-Universität Gießen, Gießen, Deutschland; 5 Institut für Neuropathologie, Universitätsmedizin Göttingen, Göttingen, Deutschland; 6 Abteilung für Neuropathologie, Philipps-Universität Marburg, Marburg, Deutschland; 7 Institut für Neuropathologie, Universität des Saarlandes, Homburg, Deutschland; 8 Institut für Neuropathologie, Universitätsklinikum Hamburg-Eppendorf (UKE), Hamburg, Deutschland; 9 Exzellenzcluster NeuroCure, Berlin, Deutschland; 10 Deutsches Zentrum für Neurodegenerative Erkrankungen (DZNE), Berlin, Deutschland; 11 Institut für Neuropathologie, Universitätsklinikum der RWTH Aachen, Aachen, Deutschland; 12 Fachbereich Gesundheit, Technische Hochschule Mittelhessen, Gießen, Deutschland

**Keywords:** SARS-CoV-2, Neuropathologie, Nervensystem, Obduktion, Register, SARS-CoV-2, Neuropathology, Nervous system, Autopsy, Registry

## Abstract

Die gesundheitlichen Auswirkungen der Coronavirus-Krankheit 2019 (COVID-19) durch die Infektion von SARS-CoV‑2 (Schweres-Akutes-Respiratorisches–Syndrom-Coronavirus 2) werden mit der Ausbreitung der Pandemie immer deutlicher. Neben der Lunge sind auch andere Organe betroffen, welche die Morbidität und Mortalität deutlich beeinflussen können. Insbesondere neurologische Symptome unter Beteiligung des zentralen und peripheren Nervensystems können akute Symptome oder Langzeitfolgen auslösen. Die Mechanismen dieser Neuropathogenese der SARS-CoV-2-Infektion und ihr Zusammenhang mit akuten und chronischen neurologischen Symptomen sind Gegenstand aktueller Studien, die sich mit der Untersuchung einer potenziellen direkten und indirekten Virusinfektion des Nervensystems beschäftigen. In der folgenden Übersichtsarbeit wird der aktuelle Stand über die neuropathologischen Manifestationen, die molekulare Pathogenese, die möglichen Infektionswege im Nervensystem und die systemischen Wirkungen zusammengefasst. Zusätzlich wird ein Überblick über das bundesweite Register CNS-COVID19 und Kooperationen gegeben, die zu einem besseren Verständnis der neurologischen Symptome von COVID-19 beitragen sollen.

## Abstract (en)

The health effects of coronavirus disease 2019 (COVID-19) caused by the infection of SARS-CoV‑2 (severe acute respiratory syndrome coronavirus 2) are becoming increasingly clear as the pandemic spreads. In addition to the lungs, other organs are also affected, which can significantly influence morbidity and mortality. In particular, neurological symptoms involving the central nervous system can lead to acute or long-term consequences. The mechanisms of this neuropathogenesis of SARS-CoV‑2 infection and its relation to acute and chronic neurological symptoms are the subject of current studies investigating a potential direct and indirect viral infection of the nervous system. The following review summarizes the current status of neuropathological manifestations, molecular pathogenesis, possible infection pathways in the nervous system, and systemic effects. In addition, an overview of the Germany-wide CNS-COVID19 registry and collaborations is presented, which should contribute to a better understanding of the neurological symptoms of COVID-19.

Eine sichere ätiologische Zuordnung der unterschiedlichen neurologischen Symptome bei Patienten mit SARS-CoV-2-Infektionen ist bisher nicht immer möglich, insbesondere, ob es sich um primär virusinduzierte, autoimmunassoziierte oder sekundäre Veränderungen handelt. Feingewebliche Untersuchungen können helfen, die zugrunde liegenden Mechanismen besser zu verstehen. Daher ist es dringend erforderlich, auch weiterhin Obduktionen von verstorbenen COVID-19-Patienten durchzuführen. Die neuropathologische Untersuchung von Gewebe aus Zentralnervensystem und peripherem Nervensystem kann hierbei wichtige Erkenntnisse zum Verständnis der neurologischen Symptome liefern und Therapieentscheidungen verbessern.

## Neuropathologische Manifestationen einer SARS-CoV-2-Infektion

### Zentrales Nervensystem (ZNS)

Eine SARS-CoV-2-Infektion kann mit einem breiten Spektrum neurologischer Komplikationen assoziiert sein [44]. In Autopsiestudien konnte gezeigt werden, dass vor allem zerebrovaskuläre Ereignisse wie ischämische Infarkte, verursacht durch Mikrothromben oder eine Schädigung der zerebralen Gefäße, auftreten können (Tab. 1). Bei Betrachtung der größeren neuropathologischen Studien [14, 37-38, 48, 51] zeigen sich bei ca. 13 % der Autopsien fokale Infarkte. Im klinischen Kontext konnte die SARS-CoV-2-Infektion als unabhängiger Schlaganfall-Risikofaktor identifiziert werden [2]. Überdies zeigt eine aktuelle klinische Metaanalyse von akuten zerebralen Schlaganfällen eine Inzidenz von 1,4 %. Dabei sind diese am häufigsten mit akuten ischämischen Infarkten assoziiert (87 %) [41]. Jedoch ist die zerebrale Hypoxie in neuropathologischen Studien nicht immer einheitlich definiert, wobei globale hypoxisch-ischämische Zustände, möglicherweise als Folge einer respiratorischen Insuffizienz bei COVID-19, von fokalen zerebralen thrombembolischen Ereignissen zu unterscheiden sind.

**Table 1:** 

Inflammation	Ischämie/Blutungen	Sonstiges	Anzahl Hirnsektionen	Literaturquelle
MG ↑ *n* = 7/7	Akute hypoxische Enzephalopathie *n* = 2/7	–	7	Deigendesch et al. (2020) [14]
Inflammation *n* = 4/18	Akute globale Ischämie *n* = 18/18	–	18	Solomon et al. (2020) [51]
MG ↑, meningeale T‑Zellen *n* = 34/43	Akute Ischämie *n* = 6/43	Astrogliose *n* = 37/43	43	Matschke et al. (2020) [37]
ADEM-ähnliche Läsionen	Teils hämorrhagisch	–	1	Reichard et al. (2020) [47]
MG ↑ *n* = 13/25	Akute Infarkte *n* = 6/33, Blutungen *n* = 2/33	–	33	Meinhardt et al. (2021) [38]
Keine gefunden	Fokale Ischämie *n* = 3/11, Blutungen *n* = 3/11	Ödem *n* = 5, Spongiose *n* = 10/11	11	Remmelink et al. (2020) [48]
Keine gefunden	Globale Ischämie *n* = 4/4	–	4	Kantonen et al. (2020) [31]
Hirnstammenzephalitis *n* = 1/2	Infarkte *n* = 2/2	–	2	Jensen et al. (2020) [29]
Keine gefunden	Infarkte *n* = 2/2	Läsionen im Marklager ähnlich zu Reichard* et al.*	2	Jaunmuktane et al. (2020) [28]
Keine MG ↑ *n* = 20/20, T‑Zellen ↑ *n* = 2/20	Infarkte *n* = 6/20	–	23	Bryce et al. (preprint) [5]
Immunzellinfiltration und MG ↑ auf Transkriptionsebene	–	–	2	Yang et al. (preprint) [57]

Studien mit mehreren Fällen und/oder neuen Befunden

*MG* Mikroglia, *ADEM* akute disseminierte/demyelinisierende Enzephalomyelitis

Entzündungen des ZNS sind nur in einzelnen Fällen in Form einer Meningoenzephalitis oder eines ADEM(akute disseminierte Enzephalomyelitis)-ähnlichen Verlaufs beschrieben. Viele Studien berichten eine „Mikrogliaaktivierung“, ohne dass immer genau angegeben wird, wie diese definiert ist und nachgewiesen wurde. Generell ist daher eine internationale Abstimmung zu Mindestanforderungen beim Bericht von „Inflammation“ und „Ischämie/Blutung“ sinnvoll, da diese Begriffe bisher zu uneinheitlich verwendet wurden (siehe Tab. 1). Dieser Umstand macht eine systematische Metaanalyse der bisherigen Daten hierzu – unabhängig von der insgesamt noch geringen Fallzahl – kaum möglich.

Da die Patienten mit COVID-19 häufig an einer bakteriellen Superinfektion und Sepsis versterben, sind vergleichende Untersuchungen mit ähnlichen Krankheitsbildern sinnvoll, fehlen aber in vielen bisher veröffentlichten Studien. Um SARS-CoV-2-spezifische neuropathologische Veränderungen zu verifizieren, sollten daher alle Befunde sowohl neuropathologisch, pathologisch als auch klinisch erfasst und miteinander korreliert werden.

### Peripheres Nervensystem (PNS) und Skelettmuskulatur

Eine Verminderung oder Veränderung des Riechvermögens gilt als ein typisches Frühsymptom bei SARS-CoV-2-Infektion und wird durch eine Infektion der olfaktorischen Sinnes-, aber auch Stützzellen erklärt [4, 36, 38]. Zudem sind COVID-19-assoziierte Guillain-Barré-Syndrom(GBS)-Verläufe beschrieben, die auch bei Patienten ohne weitere COVID-19-Symptome auftreten können [12, 44]. Ein GBS ist eine postinfektiöse Neuropathie, bei welcher Antikörper mit den Glykolipiden der Myelinscheiden peripherer Nerven kreuzreagieren und es zu einer Schädigung dieser kommt. Möglicherweise ist auch bei COVID-19 eine derartige postinfektiöse, immunmediierte Pathogenese von Bedeutung.

Erhöhte Creatinkinase(CK)-Werte, ein Marker für Muskelfaseruntergänge, wird in 27 % der Patienten mit COVID-19 beschrieben [44, 45]. Eine geringere Anzahl an Patienten zeigt sehr hohe CK-Werte >10.000 U/L als Zeichen einer nekrotisierenden Myopathie [12]. Allerdings fehlen bisher systematische morphologische Untersuchungen von peripheren Nerven und Skelettmuskulatur, die über die Häufigkeit und die Ätiologie einer neuromuskulären Beteiligung bei COVID-19 Auskunft geben können. Möglicherweise werden auch bestehende neuromuskuläre Erkrankungen durch COVID-19 verstärkt [19]. Zusätzlich kann die Beurteilung des peripheren Nervensystems und der Skelettmuskulatur durch oft lange und schwere Krankheitsverläufe mit Folgen, wie z. B. Critical-illness-Myopathie oder Critical-illness-Neuropathie, sowie durch die Wirkung medikamentöser Therapien erschwert werden [9].

## Virale Eintrittspforten und deren molekulare Mechanismen

**Figure 1:**
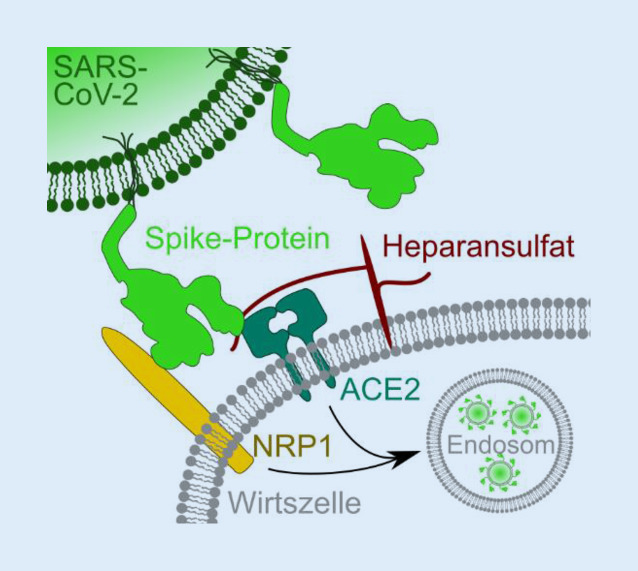

Die molekularen Mechanismen der SARS-CoV-2-Infektion auf zellulärer Ebene sind noch nicht vollständig geklärt. Es wurde gezeigt, dass SARS-CoV‑2, ähnlich dem SARS-CoV, das Angiotensin-konvertierende Enzym 2 (ACE2) als Rezeptor verwendet, an den das virale Spike-Glykoprotein bindet ([56]; Abb. 1). ACE2 könnte zwar als ein primärer Rezeptor in einigen Zelltypen des ZNS vorkommen, die mRNA-Steady-State-Level scheinen jedoch nicht mit dem Infektionspotenzial zu korrelieren, sodass möglicherweise weitere Rezeptoren als Eintrittspforten dienen. Außerdem wurde Heparansulfat als potenzieller Hilfsfaktor der initialen SARS-CoV-2-Bindung an die Zelle identifiziert [10]. Im ZNS weisen Studien auf eine Bindung von SARS-CoV‑2 an das Rezeptorprotein Neuropilin‑1 (NRP1) hin, welche die Infektiosität von SARS-CoV‑2 deutlich potenziert. Fraglich ist, ob NRP1 nur zusammen mit ACE2 oder auch als unabhängiger Rezeptor funktioniert [7, 13]. Im ZNS ist NRP1 im Gegensatz zu ACE2 in verschiedenen Zelltypen, z. B. in Astrozyten, Mikroglia oder Endothelzellen, sehr deutlich detektierbar (sowohl auf mRNA- als auch auf Proteinebene), was den Eintritt in diese Zellen erleichtern könnte [38]. Hier würden weitere Einzelzell-RNA-Sequenzierungsanalysen zur Klärung beitragen [26].

Nach der Aufnahme des Virus in die Wirtszelle ist eine Spaltung des Spikeproteins durch zelluläre Proteasen für die Fusion von SARS-CoV‑2 mit der Zellmembran nötig. Hier wurde die mögliche Beteiligung der transmembranen Serinprotease 2 (TMPRSS2), Cathepsin B und L sowie Furin gezeigt. Für TMPRSS2 wurde die dafür nötige proteolytische Aktivierung des Spikeproteins bereits mechanistisch aufgeklärt [26].

## Infektionsrouten des SARS-CoV-2 ins ZNS

**Figure 2:**
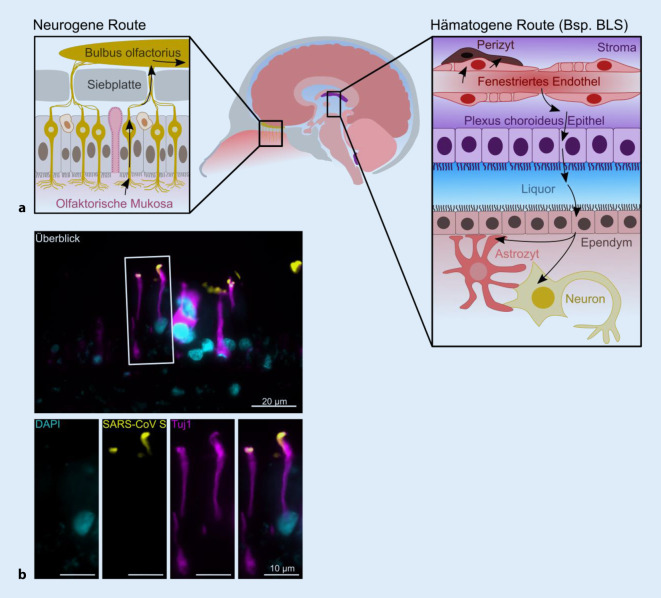

Die Invasion der Coronaviren und die Ausbreitung im ZNS kann vermutlich auf zwei Wegen erfolgen: 1. hämatogen oder 2. neurogen (Abb. 2). Darüber hinaus wird das glymphatische System, bestehend aus olfaktorischen und zervikalen lymphatischen Blutgefäßen, als Infektionsroute diskutiert [3].

### Die hämatogene Route

Viruspartikel aus dem Blutplasma können das ZNS durch rezeptorvermittelte Transzytose über die Blut-Hirn-Schranke (BHS) und die Blut-Liquor-Schranke (BLS) infizieren (Abb. 2). Da die SARS-CoV-2-Viruspartikel einen Durchmesser von ca. 100 nm haben [1], sind auch die fenestrierten Endothelien der meisten zirkumventrikulären Organe für das Virus nur durch Transzytose durchgängig. In Übereinstimmung mit einer Expression der SARS-CoV-2-Rezeptorproteine ACE2 und NRP1 in Endothelzellen konnte SARS-CoV‑2 in zerebralen Endothelzellen in Autopsiestudien nachgewiesen werden [38]. Weiterhin wird diskutiert, ob SARS-CoV‑2 zu einer Schädigung der Endothelbarrieren führt, beispielsweise durch einen systemischen Zytokinsturm [26, 27, 43]. Untersuchungen an Choroid-Plexus-Organoiden/Hirnorganoiden zeigen, dass SARS-CoV‑2 einen Tropismus für Epithelzellen des Plexus choroideus aufweist, Neurone und Gliazellen beispielsweise aber weniger deutlich infiziert [27, 43, 46]. Über die Infektion des Plexus-choroideus-Epithels ist eine Verbreitung über den Liquor in viele weitere Gehirnareale denkbar. Systematische, in Kohortenstudien reproduzierte Nachweise von viraler mRNA im Liquor oder Proteinnachweise stehen aber noch aus (Tab. 2). Darüber hinaus können Coronaviren Leukozyten infizieren, welche in ihrer aktivierten Form die BHS nach dem Prinzip des „leukocyte trafficking“ überqueren können. So könnten diese SARS-CoV‑2 als „Trojanisches Pferd“ für den Eintritt ins ZNS dienen. Für diesen Weg liegen aber bisher keine schlüssigen Daten vor [26, 43].

**Table 2:** 

Methode^a^	Lokalisation	Literaturquelle
RT-qPCR	Lobus frontalis	Paniz-Mondolfi et al. (2020) [42] Matschke et al. (2020) [37] Yang et al. (preprint) [57] Solomon et al. (2020) [51]
RT-qPCR	Liquor cerebrospinalis	Moriguchi et al. (2020) [40]
RT-qPCR	Medulla oblongata	Matschke et al. (2020) [37] Meinhardt et al. (2021) [38] Solomon et al. (2020) [51]
RT-qPCR	Ganglion trigeminale	Meinhardt et al. (2021) [38]
RT-qPCR	Bulbus olfactorius	Meinhardt et al. (2021) [38] Solomon et al. (2020) [51]
RT-qPCR	Cerebellum	Meinhardt et al. (2021) [38]
Sequenzierung	Liquor cerebrospinalis	Zhou et al. (2020) [58]
IHC	Olfaktorische sensorische Neurone (Epithelium olfactorium)	Cantuti-Castelvetri et al. (2020) [7] Meinhardt et al. (2021) [38]
IHC	N. olfactorius	Meinhardt et al. (2021) [38] Solomon et al. (2020) [51]
IHC	N. vagus, N. glossopharyngeus	Matschke et al. (2020) [37]
IHC	Cerebellum	Meinhardt et al. (2021) [38]
IHC	Gefäßendothel	Meinhardt et al. (2021) [38]
IHC	Cortex cerebri	Song et al. (preprint) [52] Yang et al. (preprint) [57]
IHC	Meningen	Yang et al. (preprint) [57] Meinhardt et al. (2021) [38]
IHC	Plexus choroideus	Yang et al. (preprint) [57]
IHC	Medulla oblongata	Matschke et al. (2020) [37] Meinhardt et al. (2021) [38]

*IHC* Immunhistochemie, *RT-qPCR* Reverse-Transkriptase quantitative Polymerase-Kettenreaktion

^a^Ein eindeutiger elektronenmikroskopischer Nachweis von SARS-CoV‑2 im zentralen Nervensystem ist unserer Meinung nach bisher noch ausstehend

### Die neurogene Route

Eine retrograde neurogene Infektion erfolgt möglicherweise über das olfaktorische System [15, 38]. Damit vereinbar ist ein vermindertes oder verändertes Riechempfinden, welches bei vielen COVID-19-Patienten auftritt [4]. Die Rezeptoren ACE2 und TMPRSS2 sind in der Nasenschleimhaut auf mRNA- und Proteinebene nachweisbar, wobei sie sich vorwiegend auf den Epithelzellen und weniger auf den olfaktorischen Neuronen finden. Dafür zeigen olfaktorische Neuronen eine deutliche NRP1-Expression [7]. Eine transsynaptische Ausbreitung nach Endozytose und axonalem Transport konnte für die humanen Coronaviren HCoV-OC43 und SARS-CoV im Mausmodell bereits gezeigt werden [15]. Daneben könnten weitere Hirnnerven (z. B. N. trigeminus) als Infektionsrouten in das ZNS dienen [38]. Obwohl erste Studien auf eine mögliche neuronale Ausbreitung von SARS-CoV‑2 im ZNS hindeuten (Tab. 2), sind weitere Untersuchungen notwendig, um diesen Weg besser zu verstehen [26].

## Indirekte und systemische Effekte von SARS-CoV-2 auf das Nervensystem

Die neurologischen Symptome und neuropathologischen Veränderungen bei COVID-19-Patienten können durch direkte Virus-induzierte und indirekte, systemische oder therapieinduzierte Effekte verursacht werden.

### Organversagen

Durch ein kardiorespiratorisches Versagen bei COVID-19-Patienten kann es zu einer globalen Hypoxie im ZNS kommen, nachweisbar vor allem in hypoxiesensiblen Arealen wie dem Kleinhirn und dem Hippocampus [26]. Oft ist noch unklar, inwieweit eine direkte Infektion der Organe eine Rolle spielt. In jedem Fall könnten systemische metabolische Veränderungen, die auf einer Funktionseinschränkung verschiedener Organe wie Niere oder Leber beruhen, zur neurologischen Symptomatik beitragen [26].

### Autonomes Nervensystem

Eine besondere Rolle im Kontext von COVID-19 kommt möglicherweise dem autonomen Nervensystem zu. Patienten mit Vorerkrankungen, die mit einer erhöhten Aktivität des sympathischen Nervensystems zusammenhängen, wie beispielsweise arterielle Hypertonie, Diabetes mellitus Typ 2 oder Herzerkrankungen, weisen eine höhere Morbidität und Mortalität auf. Diese könnten einerseits auf eine Hyperaktivierung der ohnehin übermäßig aktiven sympathischen Neuronen, beispielsweise im Hirnstamm, zurückzuführen sein. Andererseits könnte der in diesen Patienten als vermindert aktiv beschriebene neurovagale antiinflammatorische Reflex zur höheren Sterblichkeit beitragen [15]. Darüber hinaus könnte, auch bei Patienten ohne Vorerkrankungen, eine Aktivierung des Hypothalamus zur Aktivierung des autonomen Nervensystems über die Hypothalamus-Hypophysen-Nebennierenrinden-Achse und damit einhergehend zu einer Immundysregulation führen [25]. In jedem Fall ist noch unklar, inwieweit direkte oder indirekte Effekte der Infektion hier eine Rolle spielen.

### Systemische (Hyper‑)Inflammation und postinfektiöse Autoantikörper

Im Normalfall löst eine Virusinfektion eine zelluläre Reaktion mit Ausschüttung von Zytokinen (vor allem Interferone [IFN]) aus. SARS-CoV‑2 scheint diesen Mechanismus wie viele andere Viren teilweise zu umgehen [55]. Eine Studie zeigte, dass schwer erkrankte Patienten wesentlich geringere IFN-α-Level aufwiesen als leicht- und mittelschwer erkrankte Patienten. Gleichzeitig zeigte sich bei diesen Patienten stark erhöhte Werte anderer Zytokine (IL‑6, IL-10 und TNF-α). Dieses Phänomen wird als Zytokinsturm bezeichnet [21]. An seiner Entstehung sind vermutlich dysregulierte myeloide Zellen beteiligt [55]. Vermutlich führt der Zytokinsturm zur Schädigung der BHS und BLS. Somit könnten neben Zytokinen auch Virusbestandteile in Liquor und das Gehirn allgemein gelangen, welche dort in Mikrogliazellen sowie perivaskulären und meningealen Makrophagen eine Immunreaktion auslösen und den systemischen Zytokinsturm verstärken, welcher zusätzlich zur ZNS-Schädigung beiträgt [26, 38].

Darüber hinaus zeigen Studien eine reduzierte Anzahl und/oder veränderte Aktivität von Lymphozyten im peripheren Blut der Patienten, vor allem natürliche Killerzellen sowie CD4- und CD8-T-Zellen, die mit der Schwere der Erkrankung korreliert [55]. Eine andere Studie fand in schwerkranken Patienten vermehrt polyklonale GM-CSF+-CD4-T-Zellen, die so auch bei Patienten mit inflammatorischen Autoimmunerkrankungen, unabhängig von COVID-19, gefunden wurden [55]. Auch wurden in Patienten hochaffine SARS-CoV-2-neutralisierende Antikörper entdeckt, die mit Säugetierepitopen – auch im Gehirn – kreuzreagierten [32]. Diese Autoantikörper könnten auch lange nach einer durchgestandenen SARS-CoV-2-Infektion noch für neurologische Spätfolgen wie anhaltender Geruchsverlust oder ein Ermüdungssyndrom („chronic fatigue“) (mit-)verantwortlich sein.

### Hyperkoagulabilität

**Figure 3:**
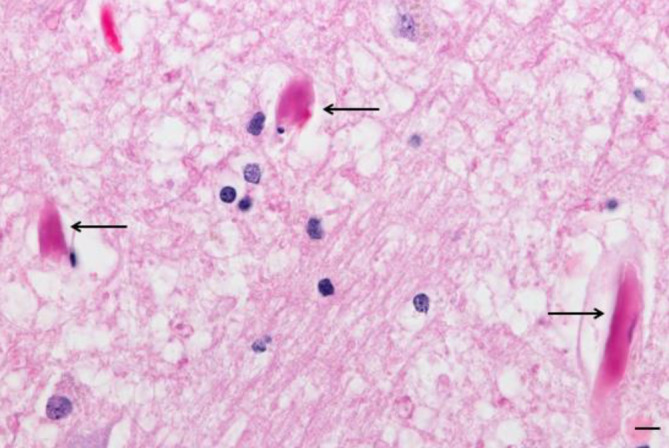

Eine erhöhte Gerinnbarkeit des Blutes (Hyperkoagulabilität), mit Embolien und Mikrothromben in einer Vielzahl von Organen, ist ein weiteres Hauptmerkmal schwerer COVID-19-Verläufe ([11]; Abb. 3). Diese Befunde sind gut vereinbar mit ischämischen ZNS-Infarkten als eine gehäufte Komplikation bei COVID-19-Erkrankten (Tab. 1). Die Pathogenese der gestörten Prokoagulanz-Antikoagulanz-Balance ist nicht abschließend geklärt. Möglicherweise spielt dabei die Aktivierung des Komplementsystems und eine vermehrte Ausschüttung von koagulationsförderndem IL‑6 sowie die Rekrutierung von Neutrophilen eine entscheidende Rolle. Mit dem Ziel, Pathogene zu immobilisieren, entlassen Neutrophile „neutrophil extracellular traps“ (NETs, extrazelluläre Netze aus kondensiertem Chromatin und antimikrobiellen Proteinen), welche Ausgangspunkt einer Thrombusbildung sein können [39]. Weiterhin tragen diffuse, endotheliale Veränderungen mit Entzündungsanzeichen wie eine erhöhte Von-Willebrand-Faktor-Konzentration zur Entstehung von Thromben bei [11]. Die bisherigen Erkenntnisse weisen darauf hin, dass sich Hyperinflammation und Hyperkoagulabilität bei COVID-19-Patienten gegenseitig verstärken.

### Therapiefolgen

Aufgrund des erhöhten Risikos von multimorbiden Patienten für einen schweren COVID-19-Krankheitsverlauf haben Patienten, die an COVID-19 versterben, häufig weitere, v. a. internistische Grunderkrankungen mit z. T. neurologischer Symptomatik, die die Interpretation der neuropathologischen Befunde erschweren. Darüber hinaus kann eine intensivmedizinische Behandlung mit Langzeitbeatmung oder extrakorporaler Membranoxygenierung (ECMO) und die extrakorporale Lungenunterstützung (ECLA) zu neuropathologischen Veränderungen am Gehirn, wie z. B. intrazerebralen Hämorrhagien, führen. Eine genaue Abgrenzung der Ätiologie durch neuropathologische Expertise ist daher zwingend notwendig, um die Ursache und Pathogenese der neuropathologischen Veränderungen bei COVID-19 zu eruieren.

## Modelle zur Untersuchung der Effekte einer SARS-CoV-2-Infektion auf das ZNS und PNS

Um die Infektions‑, Verbreitungs- und Schädigungsmechanismen von SARS-CoV‑2 im ZNS besser zu verstehen und auch potenzielle Therapien zu testen, bedarf es je nach konkreter Fragestellung geeigneter In-vitro- und In-vivo-Modelle.

### In-vitro-Modelle

Die meisten bisher eingesetzten etablierten Zelllinien, z. B. Vero E6 (Nierenepithelzellen aus grüner Meerkatze), HEK 293T (humane Nierenepithelzellen), Huh7 (aus humanem hepatozellulärem Karzinom), Caco‑2 (aus humanem kolorektalen Karzinom) oder Calu‑3 (aus humanem Lungenkarzinom), sind nicht ZNS- oder PNS-spezifisch und können daher nur begrenzt für ZNS-spezifische Fragestellungen verwendet werden [35, 50, 54]. Hingegen sind neuronale Kulturen, Neurosphären und 3D-Gehirn-Organoide aus induzierten pluripotenten humanen Stammzellen gut geeignet, um die Effekte einer SARS-CoV-2-Infektion auf Zellen des ZNS *in vitro* zu untersuchen [6, 8, 27, 43, 46, 52]. Dabei konnte die Infizierbarkeit neuronaler Progenitorzellen sowie von Neuronen und Plexusepithelzellen gezeigt werden. Über eine aktive virale Replikation in Neuronen wird dabei unterschiedlich berichtet.

### In-vivo-Modelle

Als für eine SARS-CoV-2-Infektion empfängliche Tiermodelle werden vorrangig Mäuse, Hamster, Frettchen und nichthumane Primaten (NHP) genutzt [17, 18, 24, 33, 50]. Die Suszeptibilität der Tiermodelle determiniert sich dabei durch die Bindungsaffinität zum tierspezifischen ACE2 oder der Proteaseaktivität für die Spaltung des viralen Spikeproteins. Da Mäuse aufgrund von Unterschieden zwischen murinem und humanem ACE2 *per se* nicht empfänglich für eine Infektion mit humanem SARS-CoV‑2 sind [59], werden transgene Mauslinien mit Expression des humanen ACE2 unter verschiedenen Promotoren und damit auch erweitertem Organtropismus verwendet [17, 24]. Dies führt bei *Krt18*-hACE2 Mäusen und *betaActin*-hACE2-Mäusen zu einer schweren Pneumonie mit typischer Inflammation, Thrombosen und auch Anosmie [22, 24]. Bei diesen Linien tritt bei SARS-CoV- und SARS-CoV-2-Infektionen auch eine Invasion des Gehirns und z. T. Enzephalitis auf, was bei COVID-19 in dieser Ausprägung nicht beobachtet wird. Weiterhin konnte durch eine virale transiente Transduktion eine Expression von hACE2 gezielt im Respirationstrakt erreicht werden, was zu schweren Pneumonien mit hohen Viruslasten führt, wie auch bei COVID-19 [22, 24]. Eine Adaptation von SARS-CoV‑2 an die Maus gelang durch die Adaptation der Rezeptorbindedomäne des Spikeproteins über Mauspassagen sowie die Einführung gezielter Mutationen, auch über CRISPR/Cas-Technologien, was die Verwendung von Wildtypmäusen erlaubt [22, 24]. Hierbei treten schwerere Krankheitsverläufe – auch mit ARDS – als bei hACE2-transgenen Mäusen auf.

Hamster gelten auch als geeignetes Tiermodell, um die SARS-CoV-2-Infektion im oberen und unteren Respirationstrakt mit entsprechenden histopathologischen Veränderungen abzubilden [17, 24]. Dies trifft auch für Frettchen zu, die vor allem auch für Studien zur direkten und indirekten Übertragung des Erregers wertvolle Ergebnisse liefern [17, 24, 33].

Als NHPs werden Rhesusaffen, Makaken und Grüne Meerkatzen verwendet, die meist auch einen milden COVID-19-Verlauf mit leichten klinischen Symptomen, Virusnachweis und histopathologischen Läsionen im Respirationstrakt aufweisen. Ein Zytokinsturm mit systemischer Entzündungsreaktion wie bei COVID-19 wurde jedoch nicht beobachtet. Die Infektion von alten Grünen Meerkatzen und Rhesusaffen führt jedoch zu schwereren Krankheitsverläufen und kann so auch als Modell für Altersdisposition bei COVID-19 verwendet werden [22, 24].

## COVID-19-Register

**Figure 4:**
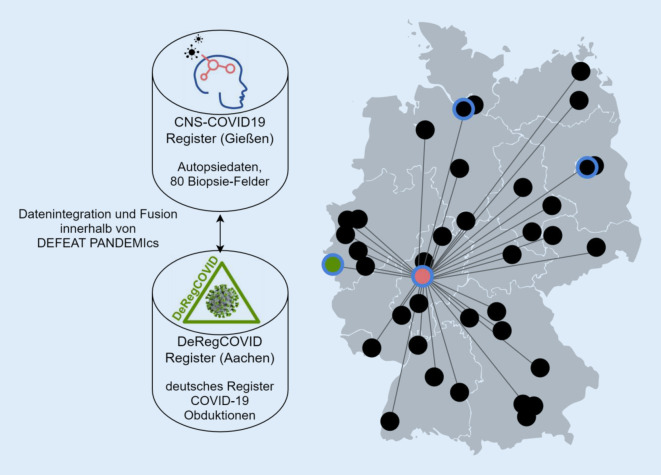

Zum schnelleren und besseren Verständnis einer Beteiligung des ZNS und PNS bei COVID-19 werden systematische Untersuchungen von Gewebeproben ausreichend großer Patientenkohorten benötigt. Aus diesem Grund hat die Deutschen Gesellschaft für Neuropathologie und Neuroanatomie (DGNN e. V.) das deutschlandweite Register CNS-COVID19 zur Zusammenführung von multizentrisch vorliegenden klinischen Daten und Obduktionsbefunden und dezentralem Biosampling gegründet. Dieses steht der wissenschaftlichen Gemeinschaft zur Verfügung und vernetzt sich mit vorhandenen klinischen Patientenregistern. CNS-COVID19 sammelt Autopsiedaten und zugehörige Metadaten von über 35 universitären und außeruniversitären (neuropathologischen) Instituten. Der bestehende intersektorale Austausch wurde im Rahmen des DEFEAT-PANDEMIcs-Projekts des „Netzwerks Universitätsmedizin“ noch erweitert (Abb. 4). Dieses Projekt zielt auf eine deutschlandweite systematische Zusammenführung von Daten und Erkenntnissen im Pandemiefall durch den Aufbau eines nationalen Obduktionsnetzwerkes ab. Extract-Transform-Load(ETL)-Strecken, die Teil eines eigens erstellten Datenintegrationssystems sind, harmonisieren die Informationen aus unterschiedlichen Quellen und vereinen sie als homogene Datenbasis im DeRegCovid-Register, einem nationalen fachübergreifenden COVID-19-Autopsieregister an der Universitätsklinik Aachen. Die Nutzung der Daten wird mittels der Infrastruktur des Nationalen Forschungsnetzwerks unter Einbindung der von der Medizininformatikinitiative (www.medizininformatik-initiative.de) etablierten Datenintegrationszentren ermöglicht (https://www.bmbf.de/de/karliczek-netzwerk-universitaetsmedizin-startet-vertiefte-forschungsarbeit-zu-covid-19-12649.html). Perspektivisch ist es sinnvoll, weitere mit Bezug auf COVID-19-Erkrankungen und damit verbundene pathologische Merkmale entwickelte klinische Register in die Datenintegration mit einzubeziehen. Das von der DGI (Deutsche Gesellschaft für Infektiologie) und dem DZIF (Deutsches Zentrum für Infektionsforschung) konzipierte „lean european open survey for SARS-CoV‑2 infected patients“ (LEOSS) sammelt europaweit Daten zu Epidemiologie und Krankheitsverlauf von Patienten mit COVID-19 ([49]; https://leoss.net). Die Studie „pooled analysis of neurologic disorders manifesting in intensive care“ (PANDEMIC) der Deutschen Gesellschaft für Neurointensiv- und Notfallmedizin (DGNI) betrachtet die Charakteristika von COVID-19-Patienten mit neurologischen Manifestationen und gleichzeitigem schweren Verlauf (https://www.dgni.de/forschung/ignite-initiative-klinischer-multizenter-studien.html). Die institutions- und fachübergreifende Zusammenführung aller Daten ist hilfreich für die Pathogeneseforschung bei COVID-19-Erkrankung und bildet eine exzellente Grundlage für den schnellen Aufbau vergleichbarer Infrastrukturen bei zukünftigen Pandemien.

### Empfehlungen zur Probenentnahme und Fixierung

**Figure 5:**
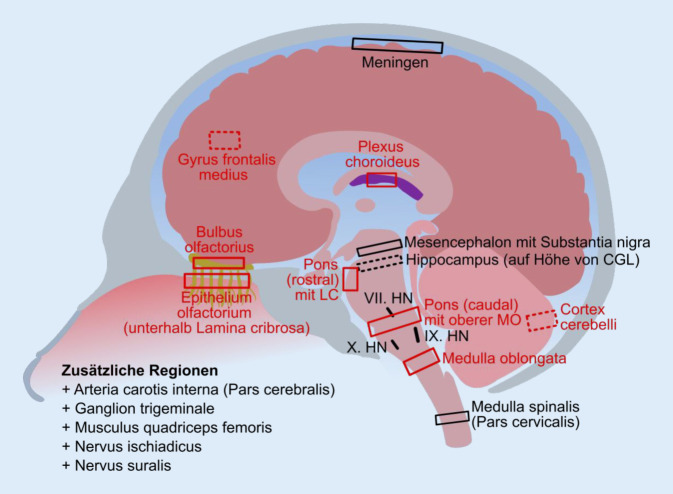

Die bisherigen Ergebnisse zeigen die Vorzüge einer Untersuchung an ausreichend großen Patientenkohorten im Vergleich zu einzelnen Fallstudien. Im Rahmen der COVID-19 Task Force der DGNN und dem Aufbau von CNS-COVID19 wurden Empfehlungen für die standardisierte Entnahme relevanter Gehirnareale sowie zusätzlich von peripheren Nerven und Skelettmuskeln zusammengestellt (Tab. 3; Abb. 5; www.cns-covid19.de). Die Gewebeproben sollten – auch zur Virusinaktivierung – ausreichend lange in Formalin^1^ [23] bzw. Glutaraldehyd für eine Semidünnschnittpräparation und ultrastrukturelle Analyse fixiert werden. Für letztere sind neben 2–6 % gepuffertem Glutaraldehyd (GA) insbesondere zur Primärfixierung auch gepufferte Gemische mit Formaldehyd (FA) zu empfehlen (z. B. 1 % FA und 2,5 % GA). Es sind kleine (ca. 2–3 mm Kantenlänge) Gewebestücke zu entnehmen, um eine gute Fixierung und Morphologie zu gewährleisten. Zur Erfassung fokaler Infektionen sollten möglichst aus verschiedenen Regionen der Organe Proben entnommen werden, welche in vielen Schnittebenen aufgearbeitet und aufmerksam am Elektronenmikroskop durchgemustert werden sollten.

**Table 3:** 

Areal	Olfaktorisch	Gustatorisch	Kardio-respiratorisch	PNS
*Epithelium olfactorium (unterhalb Lamina cribrosa)*	X			
*Bulbus olfactorius*	X			
Hippocampus (auf Höhe von Corpus geniculatum laterale)				
*Gyrus frontalis medius*				
Mesencephalonmit Substantia nigra				
*Pons (rostral) mit Locus coeruleus (oberhalb des V. HN)*			X	
*Pons (caudal) mit oberer Medulla oblongata*		X	X	
*Medulla oblongata*		X	X	
Nervus facialis (VII. HN)		X	X	
Nervus glossopharyngeus (IX. HN)		X	X	
Nervus vagus (X. HN)		X	X	
Medulla spinalis (Pars cervicalis)			X	
*Cortex cerebelli*				
Meningen (Leptomeningen)				
*Plexus choroideus*				
Arteria carotis interna (Pars cerebralis)			X	
Ganglion trigeminale				X
Musculus quadriceps femoris^a^				X
Nervus ischiadicus^b^				X
Nervus suralis^b^				X

*Kursiv* = Kerndatensatz, *steil* = zusätzliche interessante Regionen

*HN* Hirnnerv, *PNS* peripheres Nervensystem

^a^Zusätzlich Fixierung mit 6 % Glutaraldehyd (GA)

^b^Zusätzlich Fixierung mit 4 % GA

Für eine ultrastrukturelle Suche nach Viren sollten speziell Proben aus Geweben mit möglichst hoher, durch RT-qPCR nachgewiesener Viruslast gewonnen werden, ggf. auch wenn diese ein hohes postmortales Intervall aufweisen [16, 38], primär kryoasserviert oder in Formalin fixiert wurden oder bereits als FFPE(formalinfixiertes und paraffineingebettetes)-Gewebe vorliegen. FFPE-Gewebe ermöglicht es außerdem, gezielt Infektionsfoci (Nachweis mittels Immunhistochemie oder In-situ-Hybridisierung) zu stanzen und anschließend für Elektronenmikroskopie weiter aufzuarbeiten [38]. Durch Darstellung morphologisch intakter Viruspartikel kann weiterhin eine wichtige Validierung der anderen Techniken erfolgen. Additive wie Gerbsäure können die Darstellung der Spikes deutlich verbessern [34].

**Figure 6:**
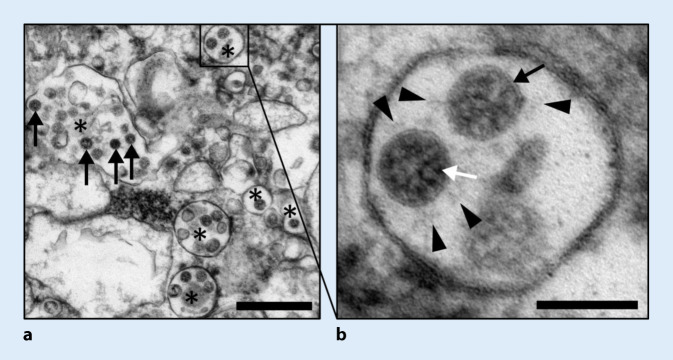

Intrazelluläre Viruspartikel (Abb. 6) sind typischerweise in Membrankompartimenten lokalisiert (Abb. 6a, *Sternchen*). Trotz starker Autolyse des Gewebes lassen sich Coronaviren gut identifizieren (Abb. 6a, *Pfeile*). Die Viruspartikel lassen sich durch ihre Substruktur mit Biomembranen (Abb. 6b, *schwarzer Pfeil*), granulärem Ribonukleoprotein (RNP; Abb. 6b, *weißer Pfeil*) und ihrer Größe von ca. 100 nm von anderen Strukturen abgrenzen. Einzelne infizierte Zellen mit dutzenden bis hunderten Viruspartikeln können als schnittinterne Positivkontrolle fungieren und so die Identifizierung von einzelnen Viruspartikeln in anderen Zellen erleichtern. Diverse morphologisch ähnliche Strukturen sollten gezielt von möglichen Viruspartikeln abgegrenzt werden [16, 25, 38]. Wichtig hierbei, vermutlich auch Grund vieler publizierter Fehleinordnungen, ist die Unterscheidung der Ultradünnschnittpräparation, in welcher die „Krone“ häufig nur angedeutet (Abb. 6b, *Pfeilspitzen*) erscheint, von der Negativkontrastierung, in welcher sie als charakteristisches morphologisches Merkmal sehr prominent ist. Weiterhin können Vesikel innerhalb von Zisternen des endoplasmatischen Retikulums, ebenfalls mit granulärer Innenstruktur, Schwierigkeiten in der Abgrenzung bereiten [25, 38]. Jedoch imponieren diese mit deutlich prominenteren und größeren Granula (17–23 nm vs. 9–16 nm) im Vergleich zum RNP der Coronaviren [25].

Da sowohl die FA- als auch GA-Fixierung zur zuverlässigen Abtötung von SARS-CoV‑2 führt [30], kann das Gewebe in einem Standard-Pathologielabor aufgearbeitet und an innerhalb von DEFEAT-PANDEMIcs unterstützte Referenzzentren versandt werden ([20]; Abb. 4). Für komplexere Methoden empfiehlt es sich, das Gewebe in Formalin zu fixieren und in Sucrose gefroren zu asservieren (ohne Paraffineinbettung) [53].

## Ausblick

Die neurologischen Manifestationen bei COVID-19 sind bisher noch unzureichend verstanden. Eine respiratorische Insuffizienz und Hyperkoagulabilität können das Risiko eines ischämischen oder hämorrhagischen Infarktes erhöhen. Es wurden bereits bei mehreren Patienten mit COVID-19 intravaskuläre Mikrothromben im ZNS nachgewiesen. Zusätzlich kann eine systemische Hyperinflammation (Zytokinsturm) diese Veränderungen verstärken. Ob und wie genau eine direkte (virale) oder indirekte Beteiligung des ZNS kausal mit einem schweren Krankheitsverlauf von COVID-19 zusammenhängt, kann erst nach systematischen neuropathologischen Untersuchungen größerer Patientenkohorten beantwortet werden. Hinweise auf eine SARS-CoV-2-Infektion in der Medulla oblongata deuten auf eine mögliche Rolle bei der kardiorespiratorischen Insuffizienz hin, da hier wichtige Nervenkerne zur Regulation des Atemzentrums und CO_2_-Chemorezeptoren lokalisiert sind. Ebenfalls könnte eine virale Infektion die Funktion spezifischer O_2_-Chemosensoren im Glomus caroticum beeinflussen. Weitere wichtige Fragen sind noch nicht abschließend geklärt. Welcher Mechanismen bedient sich das Virus, um ins ZNS zu gelangen? Wie ist die Pathogenese der beschriebenen ZNS-Schädigungen? Sind sie direkter Natur oder auf die systemische Reaktion des Körpers zurückzuführen? Während es schon verschiedene Studien zu COVID-19-Auswirkungen auf das ZNS gibt, fehlen systematische morphologische Untersuchungen des peripheren Nervensystems und der Skelettmuskulatur fast gänzlich. Zur Beantwortung der genannten Fragen kommt in der Grundlagenforschung und translationalen Forschung der Weiterentwicklung geeigneter In-vitro- und In-vivo-Modelle eine wichtige Rolle zu. Insbesondere eine systematische Erfassung von Gewebe- und Autopsieproben klinisch gut charakterisierter Patientenkohorten, die Entwicklung standardisierter Gewebeasservierungsprotokolle bei Autopsien unter Einhaltung der besonderen Hygienevorschriften sowie die Entwicklung und Etablierung von Methoden zum feingeweblichen Virusnachweis und „deep phenotyping“ werden essenziell sein, um die Rolle der Beteiligung des ZNS und PNS für den Krankheitsverlauf bei COVID-19 näher zu charakterisieren, neue Behandlungsmöglichkeiten zu entwickeln und Langzeitfolgen abzuschätzen.

## Fazit für die Praxis

- Patienten mit COVID(coronavirus disease)-19-Erkrankungen können eine Beteiligung des zentralen Nervensystems (ZNS) und des peripheren Nervensystems (PNS) aufweisen, die einen Einfluss auf Morbidität und Mortalität hat.
- Gehäuft treten hypoxämische Veränderungen inkl. ZNS-Infarkte auf, welche vermutlich durch Mikrothromben bei Hyperkoagulationssyndrom und Zytokinsturm verursacht werden.
- Ein direkter Virusnachweis von SARS-CoV‑2 („severe acute respiratory syndrome coronavirus 2“) im ZNS konnte mithilfe immunhistochemischer Methoden und molekularer Analysen erbracht werden. Als Eintrittspforte in das Gehirn werden neurogene oder hämatogene Ausbreitungswege diskutiert.
- Zum besseren Verständnis der Pathogenese der Beteiligung des ZNS und PNS bei COVID-19-Patienten müssen Gewebeproben ausreichend großer Patientenkohorten untersucht werden. Eine autoptische Untersuchung und systematische Erfassung von an/mit COVID-19 verstorbenen Patienten in einem Register ist daher dringend erforderlich.
- Systematische neuropathologische Untersuchungen des PNS und der Skelettmuskulatur fehlen bisher.

## Footnotes

1 10 % gepufferte Formalinlösung, enthält 4 % Formaldehyd (FA): mind. 48 h bei Paraffineinbettung (die übliche Paraffineinwirkung von mind. 2 h bei 60–65 °C verstärkt die Virusinaktivierung); ohne Hitzeeinwirkung besser längere Fixierungszeit [21]. Das Center for Disease Control der USA empfiehlt 72 h in 10 % gepufferter Formalinlösung (https://www.cdc.gov/coronavirus/2019-ncov/hcp/guidance-postmortem-specimens.html).
